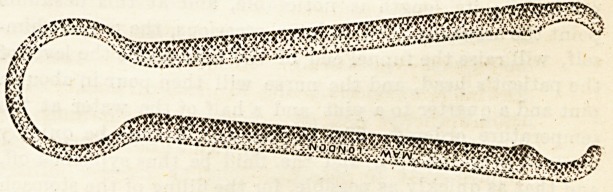# "The Hospital" Nursing Mirror

**Published:** 1898-02-12

**Authors:** 


### "The Hospital" Nursing Mirror.

Being the Nursing Section of "The Hospital."

[Contributions for this Section of "The Hospital" should be addressed to the Editor, The Hospital, 28 & 29, Southampton Street, Strand, London, W.C., and should have the word "Nursing" plainly written in left-hand top corner of the envelope.]

### News from the Nursing World.

### THE QUEEN'S VISIT TO THE RIVIERA.

The Queen's health is undoubtedly much benefited by her annual visit to the South of France, where the air suits her admirably. The west wing of the Excelsior Hotel Regina at Cimiez, containing about seventy rooms, is rented by the Queen, and is furnished by her with simple elegance. As the hotel stands high, the view from the private apartments, and especially from the balcony of Her Majesty's sitting-room, is very extensive. A garden is also set apart for her private use; here she breakfasts at ten o'clock, and twice during the day she enjoys long drives in the beautiful private grounds in the neighbourhood thrown open to her. The drainage, always troublesome in the Riviera, has been satisfactorily arranged in the hotel, so that there is no danger of an outbreak of typhoid when Her Majesty takes up her abode. She will leave England on March 9th, and remain abroad until the end of April. The Princess Beatrice accompanies Her Majesty, and will occupy rooms immediately over hers, whilst her children will go to the Villa Liserb.

### THE QUEEN'S VISIT TO NETLEY.

The Queen has expressed her intention of visiting the soldiers at the Netley Hospital on Tuesday next. Arrangements have been made for Her Majesty to cross from East Cowes to Southampton Royal Pier in the Royal yacht, and continue her journey by special train. Her visit, which will be of a strictly private character, will last about an hour, and is anticipated with great pleasure by the sick soldiers.

### A ROYAL PATRONESS.

Dr. Barnardo's enormous work amongst the waifs and strays of the metropolis has many sympathisers. It is not surprising to find the Princess of Wales amongst their number, as she takes so keen an interest in all that tends to ameliorate the condition of the poor. H.R.H. has just shown her appreciation of Dr. Barnardo's efforts on behalf of destitute children by becoming a patroness of the Young Helpers' League.

### THE HOME OF REST, SHANKLIN.

The Princess Beatrice has been very busy visiting the various hospitals in the neighbourhood of the Isle of Wight. On the 7th inst. she spent the afternoon at the Home of Rest at Shanklin. This home was given by the late Mrs. Harvey for the members of the Hampshire Girls' Friendly Society, and cost £12,000.

### THE NURSES' CO-OPERATION.

The seventh annual report of the Nurses' Co-operation shows the position and development of the society to be eminently satisfactory. The number of trained nurses on the staff is 444, the number of cases nursed reached the large total of 5,083, and the earnings amounted to a gross total of £36,800. The Princess Louise, Marchioness of Lorne, whose sympathy with all nursing enterprise is well known, has consented to be patroness. During the year Miss Philippa Hicks resigned the office of lady superintendent on account of ill-health, and her work was taken by Miss Amy Hughes, who has already given evidence of her zeal in her new and mportant position. Candidates desiring admission to the staff are elected in the first week of May and November, and may obtain particulars at 8, New Cavendish-street, W. The aim of the Co-operation is to secure to nurses their full fees, a deduction of 7½ per cent. being made to meet expenses.

### NURSES ON LEAVE FROM INDIA.

Ladies working in the military nursing service are entitled to a year's leave at the conclusion of one period of service if they engage themselves for a further term at the end of it. Regulations have just been made that the year's furlough may be lengthened to two on production of a medical certificate stating that the extension is desirable, and that the applicant will in all probability be able to resume her work at the close of it. Pay during the second year's leave of absence is wo-thirds of that allowed during the first year.

### NURSING IN THE PUNJAB.

The ravages of enteric fever amongst our soldiers are becoming more and more devastating, and the need of meeting it in the best way is pressing heavily, not only upon the authorities in India, but upon the relatives in England who lose their sons not for their country's welfare, but from a disease that can be stamped out by strict hygienic measures. Naturally the first demand in these cases is for skilled nurses, and to supply them the Up-Country Nursing Association has formed a branch to work in the Punjab. Lady Mackworthy Young is the president of a small committee of ladies and gentlemen at Lahore which will perform all administrative duties. It is proposed to secure a home at the Hill Station of Kusowlee for the nurses when not on duty and a competent matron is already engaged. The association will begin with a staff of three nurses, and we hear that such good progress has been made in collecting the necessary money that Mrs. Shepperd, 10, Chester-place, Regent's Park, has been asked to select two nurses at once. Candidates must be Protestant ladies of earnest religious principles, and possess high nursing qualifications. A local committee has been formed in England in order to raise about £400. If this amount can be remitted to India by the 15th inst., the home will be opened early in March. Persons who have relatives in the Punjab, and who may be desirous of helping the association on that account will best do so by forwarding a donation to any of the following gentlemen: Major-General Mercer, "Aldie," 13, Woodville-road, Ealing; Surgeon-General de Renzy, C.B., 20, Parkhill, Ealing; and C. J. Michôd, Esq., 1, King's-avenue, Ealing.

### GUY'S NURSES FOR AFRICA.

Nurse Nutt, Nurse Powell, and Nurse Clarke accompany Major Lugard's expedition to Africa as nursing sisters. The engagement is for one year.

### NEWS OF AN OLD FRIEND.

Rukhmabai, the Indian lady doctor, who is rendering such valuable help both to the people of her own country, and also to the English plague committee at Bombay, is an old friend of the English-speaking world. Some years ago the story of her wrongs, and the courageous way she faced them, aroused a storm of sympathy and indignation in every quarter of the globe. When she was a child of eleven she was betrothed to a youth of nineteen, of dissolute habits and repulsive appearance. Until she was twenty-two she managed to escape the fulfilment of the marriage contract, but then her legal husband called in the law to compel his unwilling bride to do so. The Judge, before whom the case was tried, refused to enforce such a barbarity; but, as his judgment was not in accordance with Indian law at that time, and a higher court reversed his verdict, Rukhmabai was threatened with six months' imprisonment for contempt if she persisted in her refusal. The affair was compromised by buying off the husband for a good round sum, and the escaped victim came to England, studied at the London School of Medicine for Women, and took her medical degree. She is now using her liberty and knowledge in the service of humanity.

### THE VALUE OF SMALL SUBSCRIPTIONS.

The Ely Nursing Association is to be congratulated on its sound financial status. This is the result of Miss Muriel's (the hon. secretary) indefatigable work in extending the subscription list amongst those who give 2s. 6d. and 5s. yearly. There is a balance to the good, and the nurse's work is satisfactory. The association has altered the rule which fixed the annual meeting in February to one permitting of its being held early in the year; this makes it possible for the committee to consult the Bishop's convenience in arranging the date, as he likes to be present.

### MRS. PARTINGTON.

The Guardians of Rochford Rural District Workhouse have trouble in obtaining nurses. The root of the difficulty appears to be that they do not want to pay them as much as other unions do. One Guardian is reported to have remarked "that a few years ago one old lady was enough; now it seems three nurses, wardsmen, and women, and a night nurse are required. It is surely time for the board to put its foot down, for surely one nurse and an assistant nurse were sufficient." Two things strike us in reading this report. First, how was the place nursed when the old lady had things all her own way? and, secondly, is not the desire to limit the nurses to two, when it is obvious that more are required, uncommonly like Mrs. Partington's determination to stop with her broom the waves of the Atlantic invading her premises?

### NURSING IN CEYLON.

The Ceylon Nursing Association is four years old, and is a remarkably healthy and useful society. Up to 1893 the number of deaths resulting from the lack of trained nursing was heartrending, and the isolated efforts to secure good nurses failed. Then four ladies met in consultation, and sent out collecting lists amongst the European residents in the island, obtaining such cordial support that they were able to engage a fully trained nurse at once. She began work in January, 1894. In June the same year another nurse was engaged, and a third in the following June. In the meantime the appreciation of the services of the Association was such that the consent of the colonel of the regiment stationed at Colombo was obtained for the employment of regimental sick orderlies, for cases to which it was impracticable to send a nurse. Other nurses employed on the island kept the hon. secretary informed of their engagements, and she gave them work when she could. In June, 1896, the need of a matron made itself so keenly felt that this post was offered to the senior nurse, who accepted it, and entered on her duties on January, 1897, a fourth nurse having been added in the previous month. At the beginning of the movement the proprietors of the Hatton estate built a nice little bungalow, with accommodation for four nurses, and leased it at a moderate rental to the association, who afterwards were able to buy it. The object of the Jubilee memorial fund, which has been most popular, was to pay for the wards just added to the nurses' home; to add another ward and sitting-room, and furnishing the same; and to pay the balance of the mortgage on the buildings. Subscribers have the privilege of a nurse at the fee of R.25 to R.30 a week, but, of course, the fees to non-subscribers are much higher.

### THE BULAWAYO HOSPITAL.

The superintendent sister of the Bulawayo Hospital has a special fund for supplying the patients with extras and dainties, and concerts and theatrical performances are held from time to time by the Bulawayoans to provide money for the purpose.

### SHORT ITEMS.

The annual members' meeting of the Nurses' National Total Abstinence League was held on January 24th at 9, Taviton Street, by kind permission of Mrs. T. P. Whittaker. Members of the nursing profession were cordially invited to join the league, meetings of which are held at seven p.m. on the first Wednesday in each month at the W.T.A.U. offices, 4, Ludgate Hill, E.C.—The Bideford and District Nursing Association are lucky in obtaining a donation of £30 from the Bideford Friendly Societies and Cyclist's Carnival Committee. The nurses are much liked. The subscriptions amounted to £50, the expenditure to £72, and there is a balance in hand of £20.—The Society for Promoting Christian Knowledge has made a grant of Prayer-books and Bibles to the Oxygen Home, in Fitzroy Square, at the request of Miss Butler.—A lady doctor from the University of Zurich has been appointed physician to the household of Menelik, the King of Abyssinia. —The Little Sisters of the Poor have a home for old and infirm poor folk at Gilmore Place. Their funds are frequently very low and always precarious. On Friday, January 28th, a successful concert was given on their behalf at the music hall, George Street, which was well attended.—The Guardians of Wandsworth and Clapham have just passed a resolution sanctioning the erection of a nurses' home at the infirmary, for which the plans have already been approved by the Board. It will cost £11,400, and arrangements will be made to borrow the money.—Miss Strangman, who has been for some time clinical assistant at the Cork Lunatic Asylum, was appointed, at the last meeting of the Board of Guardians, assistant medical officer.

### Lectures to Surgical Nurses.

By H. A. LATIMER, M.D. (Dunelm), M.R.C.S. of Eng., L.S.A. of London, Consulting Surgeon, Swansea Hospital; President of the Swansea Medical Society; Lecturer and Examiner of the St. John Ambulance Association, &c.

### XXIV.—THE HEALING OF ULCERS—BED SORES

As you watch the ulcer healing from day to day, you will notice the transparent margin push its way from the circumference of the sore towards its centre; it will not advance with perfect regularity; here and there little processes will shoot forwards as if in a hurry to finish the good work they are engaged in doing, and as they are approaching each other from different directions they eventually meet and join. In this way the granulating surface of the ulcer becomes divided into several surfaces, shut off from each other by intervening layers of this new material; and, as the whole process begins afresh in each of these separated areas, before long all the granulations are covered with it. Now the ulcer is healed. The transparent covering which I have been telling you of is new skin, formed by an extension and new growth from the outermost layer of the adjoining skin. An open wound can only finish healing in this way. Whether its edges adhere; whether the breach in continuity is filled by granulations; or whether these granulations unite with each other, the final result is the same—the line of division or the open surface has to be covered by a layer of tissue, "scar tissue," derived from the skin. Surgeons, in order to hasten healing processes in wounds, avail themselves of this fact by imitating nature. Taking minute pieces of skin from elsewhere, they apply them to the granulating surfaces, and then cover them with protective material to ensure their being undisturbed. If all is doing well when the protective material is removed, in two or three days' time the new bits of skin seem to have disappeared, but shortly afterwards the transparent tissue I have been telling you of shows itself, and a "centre of healing" has been established. These bits of skin are called "grafts," and the operation is known as "skin grafting."

A stationary ulcer has generally a surface of flabby big granulations, and its edges are raised above the adjoining skin, and are hard like gristle and insensitive; the discharge from it is thin and scanty. An ulcer which is extending can be plainly seen to be growing wider in surface, or to dip down into the parts in which it lies as the days pass by. It is often covered with a layer of sloughing tissue, and may have a bloody secretion oozing from it; or it may have an angry-looking, glazed, and red appearance. The points concerned in healing these ulcers are: Attention to cleanliness; rest of the affected part; the maintenance of proper position so as to favour circulation of blood, and to prevent its stagnation at the sore; and the application of suitable remedies, as ordered by the surgeon in attendance. These applications vary with the experience of the attendants; each man has his favourite remedies. Nowadays they mostly consist of some variety of antiseptic lotion or ointment, assisted by careful bandaging of the limb in which the ulcer occurs, whereby the circulation of the blood may be helped.

The ulcer which especially concerns nurses—and which might well be called "the nurses' ulcer"—is that known as a bed sore. I must be careful to say that in calling a bed sore "the nurses' ulcer" I do so on account only of the supreme interest and importance with which it is invested to her. My experience has taught me to reject the extreme view which I have known some men adopt of always blaming a nurse when a bed sore forms, for I have seen such a thing take place with extreme rapidity in special cases, and in low and prostrate conditions, where the bed-sheets have been constantly wetted with discharges passed by a patient. I suppose the extreme view is taken as a warning to nurses that they must unceasingly guard against the formation of bed sores; and it has a good effect, for, in the majority of cases, skilful and unremitting attention to the parts on which these are liable to form does succeed in protecting a prostrate person from this complication of illness. The parts most liable to break down into bed sores are those on which pressure is exerted on skin and the underlying flesh which cover bone. Three factors are at work in this direction: withdrawal of healthy stimulation by interference with the natural flow of nerve-force, which is necessary if the vital processes are to be carried on properly in the body; stagnation of blood leading to a sodden condition of tissues which renders them unresistant to injurious influences; and pressure between two hard surfaces—the bed and the underlying bone. By the first two conditions the body is rendered feeble, and is deprived of its power of resisting injuries; by the third the injury is inflicted.

From what I have just been saying you will be led to expect that there are certain regions in the body especially liable to the formation of bed sores, and your expectation will be a right one. These regions are: The lower part of the back, where there is but a small amount of padding over the sacrum bone at the end of the spinal column; the points over the hip bones; and the heels. The most frequent seat of all is the back one, for here not only is pressure exercised by the weight of the body—at its greatest when people are lying prostrate in bed on their backs—but also fluids of various kinds make their way along the level or inclined surface of bedding to that region, and add one more potent factor to the other injurious ones. In the majority of cases bed sores are absolutely preventable by nurses, but in order that they may be warded off they must exercise constant vigilance. In all continuous illnesses the nurse must be on the look-out for them, for she can prevent their formation, but will find it very difficult to cure them if they have been allowed to make their appearance. If the patient is conscious and in a condition to feel pain, and anything is pressing on the back or elsewhere, he will complain of feeling something in the bed, such as rucked-up sheets or crumbs lying about. And if anything is going wrong and there is a tendency to sloughing in the back, the skin at the threatened region will assume a dusky red or purplish look highly significant of congestion of blood. Twice a day at least you must actually look at the back in all illnesses where people are lying prostrate, and you must do this oftener—very much oftener—where there is incontinence of the bladder or of the rectum. If you do this you cannot fail to detect the earliest sign of things going wrong.

### Novelties for Nurses.

### COAL TONGS FOR THE SICK-ROOM.

Messrs. S. Maw, Son, and Thompson, of 7, Aldersgate Street, the well-known instrument makers, have prepared a most useful addition to the comfort of a sick-room in the form of noiseless coal tongs. The "MacMillan Tongs," as they are called, are covered with asbestos cloth, which is non-inflammable. This invention really fills a want, and is much more convenient than a glove, which, after use for a time, gets dirty and soils the hands.

### Post-Graduate Clinics for Nurses.

By a Trained Nurse.

XLII.—LAVAGE OF THE STOMACH (*continued*).

The nurse should make her preparations with as little fuss as possible, for even when the patient is used to the process it must always be suggestive of unpleasant conditions. Sterilised water may be ordered, but if not the nurse should take care that it has been boiled for at least ten minutes. About a gallon of water is the usual quantity to employ for each washing, and the temperature ranges from 100 to 103 deg. Fahr. But the doctor should invariably be consulted on this point. Four jugs, each holding about a quart, should stand ready with the carefully measured quantity of water for each lavage, a white earthenware bedroom pail making a nice receptacle for the water which is syphoned back. A large mackintosh will be needed. If the lavage is likely to be continued it is a good plan for the nurse to shape the mackintosh into a cape such as hairdressers use for their clients. A supply of soft bath towels should also be at hand. The stomach tube completes the list of necessary articles. If medication of the water be ordered, the specified quantities should be thoroughly mixed with the water before the process begins. An alkaline or antiseptic solution may be ordered, but this of course the nurse will ascertain from the doctor. It is far easier to perform lavage when the patient is sitting in an easy chair, but this position is not suited to all conditions. If unable to get up the patient needs to be well supported with a bed-rest carefully pillow-packed.

In most cases where a patient is able to sit up, I find an old easy chair, with the legs removed, put into the bed makes the most delightful and comfortable bed-rest and lounge. All nurses in private practice should bear in mind this little suggestion, and try it on the first opportunity. All preparations being made ready, one of the patient's friends should stand at his back and firmly support the head, which should be inclined a trifle backwards, but not too much so, since human beings are not accustomed to swallowing with the head in this unnatural position. Inclining the head too far back is frequently the cause of much difficulty in passing the stomach tube. An infinitesimal quantity of almond oil, as I pointed out previously, smeared on the tube greatly facilitates matters. The nurse, standing to one side of her patient, takes the tube between her thumb and index finger, and calmly and without hurry or fuss passes it over the tongue and down the œsophagus. If the nurse has an inspiring and confident manner, very often the tube will go down easily and at once, but if she hesitate and pass the tube in an uncertain manner, or if the patient be a nervous subject, an involuntary contraction of the throat will prove an obstacle in the passage of the tube. There must be no hurry. If the patient set up a paroxysm of choking, it is better not to withdraw the tube unless obliged. By waiting a few seconds the throat may accommodate itself to the foreign body, for of course the tube is a foreign body, and it is a natural proceeding on the part of the throat to attempt to expel it. When the tube reaches the stomach a slight sound of air passing up its length is noticeable, and at this desirable point the assistant, and on later occasions, the patient himself, will raise the funnel end of the tube above the level of the patient's head, and the nurse will then pour in about a pint and a quarter to a pint and a half of the water at the temperature ordered. The funnel should then be quickly lowered over the pail, and the fluid be thus syphoned off, and that as quickly as possible, for the filling of the stomach often causes an urgent desire to vomit, and in some cases this would cause unnecessary discomfort, and in every instance would spell failure so far as the nurse is concerned. After the stomach contents are syphoned out, the tube is again raised, the stomach re-filled, and again syphoned, the process being repeated till the full quantity has passed in and out of the stomach. Some doctors direct the nurses to teach their patients, when the stomach is being emptied for the last time, to place their hands on either side of the stomach region and make pressure so as to help in the removal of the gastric contents. But nurses should never do this on their own responsibility, since there are many cases, for instance, where much pain, irritation, inflammation, or ulceration is present, when much harm might be done by such a practice. All that now remains to be done is to place the patient in a comfortable horizontal position, save the syphoned liquid for medical inspection, and having carefully sterilised the stomach tube for future use, place this in a covered vessel filled with sterilised or boiled water.

### Appointments.

### MATRONS.

Royal Derby and Derbyshire Nursing and Sanitary Association.—Miss Matilda Atthill has been appointed District Superintendent in the above association. She trained at Guy's and Addenbrooke's, and qualified for her L.O.S. at the City of London. She worked for some years in connection with the Mildmay Institutions, during which time she held for three years the post of sister over the female wards of the Jaffa Hospital, and has had much experience in private and district nursing.

The New City Isolation Hospital, Worcester.—Miss Elizabeth Bowen was appointed Nurse-Matron of this hospital on February 1st. She was trained at the Liverpool Royal Infirmary and was afterwards nurse at the Infectious Hospital of Ormskirk Union. She has since been charge nurse for three years and assistant matron for one at the Worcester General Infirmary.

The Convalescent Home (Attached to the Hospital for Sick Children, Great Ormond Street), Highgate.—Miss A. E. Mandall Bell, who was trained at the Children's Hospital, Pendlebury, has been appointed Matron of this home. Miss Bell has held the post of sister of the infectious block of the Hospital for Sick Children, Great Ormond Street, for seven years.

Memorial Hospital, Kendal.—Miss Evelyn Hurlbatt was appointed Matron of this hospital on the 1st inst. She received her training at Guy's Hospital and afterwards held the post of matron to the Keynard Cottage Hospital, near Gainsboro', for two years. She has latterly taken temporary sister's duties at Guy's Hospital.

Miss Mary Leigh Swift, who was trained at Guy's Hospital, has been appointed Matron of the Albany General Hospital, Grahamstown.

### Minor Appointments.

City Hospital, Coventry.—Nurse A. E. Gillespie has been appointed Charge Nurse at the City Fever Hospital, Coventry. She was trained for three years at Fir Vale Infirmary, Sheffield, and holds the certificate of the L.O.S. diploma. Nurse Gillespie was appointed on December 10th, and commenced her duties at Coventry on February 1st.

East London Hospital for Children, Shadwell.—On February 4th Miss Maud Biggott, who was trained at Bristol Royal Infirmary, and who has been sister of the children's ward of the above hospital for the last two years, was appointed Night Sister of the same.

St. Saviour's Union Infirmary, East Dulwich Grove, S.E.—Miss Florence Wooll, who was trained at Guy's Hospital, and who has been sister at the Alexandra Hospital for Children, Brighton, has been appointed Sister at the above infirmary.

Kingston-upon-Hull Workhouse Hospital.—Miss Eva Beck was appointed Superintendent Nurse of this hospital on February 7th. She has up to the present been charge nurse of the hospital, and was trained at the Workhouse Infirmary, Birmingham.

St. Mark's Hospital for Fistula, City Road, E.C.—Miss Mary Glenton Kerr, who was trained at the Leeds General Infirmary, has been appointed Day Nurse Sister of the men's wards.

### Nursing in Paris Hospitals.

### A.—LAY NURSES.

### I.—The Campaign for their Introduction before 1878.

As all in any way familiar with hospital matters are aware, during the last twenty years there has taken place in the internal economy of the ancient hospital system of Paris a revolution, in its own small way, greater, more fundamental and far-reaching than was the political cataclysm in France of a century ago. When the full tempest of 1789 had blown over, though so many venerable foundations were either swept away for ever or were so altered that their founders would never have recognised their handiwork, the hospital system was left practically unchanged. Another century had to elapse before the revolutionary era of the hospitals was to come. It has come at last, however, and I will try to give an impartial account of what led to the change, how it has been carried out, and the comparative merits of the old and of the new. This is no easy task. It is difficult for an English or any other foreign critic to keep entirely free from the prejudice which taints all the statements, statistics, and arguments on the respective merits of Sisters of Charity and lay nurses in hospitals. Even the historical portion of the subject is given by each side with bias, and with no agreement. One side asserts that Louis XIV. was a laiciser, while the other as strenuously declares that the Sun King had no such stuff in his thoughts.

This point, at any rate, is beyond dispute—that, just two centuries ago, Louis the Great, prompted by the genius of Colbert, laid the foundations of the whole modern hospital system of France by the edict of December 12th, 1698, which first brought the hospital system of Paris, as of other French towns, into one financial control, semi-clerical and semi-lay, guaranteed by the State. The advocates of the lay system say Louis did this to curb the clerical domination; the advocates of the religious system reply that the clerics were left in charge, and some abuses only were remedied.

Louis XIV. had in 1673 restored the Hôtel Dieu to clerical control, it having been taken out of the hands of the Chapter of Notre Dame, its original owners, way back in 1505, by the Paris Parliament. The active work of nursing continued to be done, as it always has been done, by lay persons. There has been everywhere a great misapprehension on this point, which I will write upon later. Doubtless the enthusiastic and thoroughgoing disciples of the earlier days of monasticism did the disagreeable drudgery of hospital work themselves, but it has never been the same in any general sense with modern nursing nuns in Paris hospitals. The Sisters only have done the work of matron-nurses, the great army of nurses being laics.

The English hospital critic, in studying the Paris system, must always keep in view the two remarkable contrasts to London which are the result of the action of Louis XIV. The Paris hospitals are Government institutions (even most of those of private foundations), and they are all subject to certain uniform administrative regulations. Although the dominant government opinion in France since 1878, especially that represented by the crusaders of laicisation, is always prone to assert that all good things have originated since 1789, and that nothing now tolerated antedates that sacred year, this is not always, I fear, true. Thus, in the official statement* that before 1789 the hospital establishments of Paris were by no means subject to one single authority, and three several directions are instanced. This is rather a strained statement of the case. In fact, the edict of Louis XIV. for ever divorced them from independent ecclesiastical control. It was the scandal of misuse of charitable funds for hospital service which occasioned this action, just as 42 years before he had tackled the same abuse in the matter of poor relief and mendicity by the founding of the Salpêtrière.

This was the state of affairs at the French Revolution; and although, of course, during the Reign of Terror the ecclesiastical element was temporarily eliminated, it soon was reinstated, and remained in office almost intact until 1878. Some pretty crumbs of historic support are picked up by the laicisers in the fact that during the Restoration the commissary clerks of various hospitals, in Paris were changed from clerics to laics, and again during the July monarchy that certain hospitals, such as the lying-in and convalescent ones, were laicised. However, the year 1878 found the whole bulk of Paris hospitals served as mostly for ages past by monastic matrons.

In 1878, with the fall of President MacMahon, the chief power in France came into the hands of the Republicans, in place of a Monarchical Coalition, which had only continued to tolerate a nominal Republic because they could not agree as to the exact form of Monarchy to substitute for it. Rightly or wrongly, we need not decide which, the triumphant Republicans of 1878 attributed the mainstay of their opponents to clericalism. Moreover, they attributed the humiliating national disasters of 1870 to the baneful clerical influence exercised through the person of the Empress Eugénie. For these reasons the watchword of the new power was Gambetta's "*Clericalisme, voila l'ennemi,*" which was, after all, only a new version of Voltaire's "*Ecrasez l'infame,*" of a century before.

While educational and other public institutions were being secularised, the hospitals were also made additional fields for the new crusade, Paris, of course, leading the van. The hospitals of Paris, as the other municipal institutions, are financially controlled by the Municipal Council, but are under the veto of the National Government, for in modern centralised France there is no such thing known as independent local government. The Paris Municipal Council was rampantly Republican and secularist, and entered with ardour upon the campaign for laicisation. As early as 1875 the council voted in favour of the new Tenon Hospital at Ménilmontant, above Père Lachaise, being entirely laic. The Council of the Assistance Publique, however, ignored this demand. In 1877 the laicisation movement was taken in charge by Dr. Désiré Magloire Bourneville, known as the "Apostle of Laicisation," who was that year elected a member of the Municipal Council for that ancient stronghold of monasticism, the Quartier Saint Victor, and who continued a member

* "Administration Général de l'Assistance Publique à Paris." 1887. P. 1.

until 1882, when he was elected a Deputy for the Department of the Seine.

However the Municipal Council might agitate, while the national executive was in Conservative hands there was no possibility of the disturbance of the Sisters. The Council of the Assistance Publique of Paris is controlled by the Government, and, of course, up to 1878 was not of a character to sanction any attack on the religious element. Thus the year 1878 found over six hundred Sisters of Charity of different orders still installed as the superintendent nurses in the hospitals of Paris.—E. R. SPEARMAN.

### The Relations of the Public to the Nurse.

By E. STANMORE BISHOP, F.R.C.S.Eng., Hon. Surgeon Ancoats Hospital, Manchester.

(*Continued from page* 167.)

SOMETIMES, I am sorry to say, the doctor in attendance is the cause of the misunderstanding. We will suppose a really very common occurrence. The mother of a family—consisting of two sons and a daughter, the latter about twenty years of age—finds a certain amount of pain in her breast. She consults the doctor, and the daughter, who goes with her, tells him that the patient is very nervous, and that he must do all he can to minimise whatever the trouble is. The patient herself evidently is very nervous. She knows her own family history; is aware that her own mother died of cancer; has probably heard from garrulous neighbours all kinds of dreadful stories about cancer, and horrible operations for its relief. The doctor examines the part and finds that it is cancer, and that the only advice worth anything to his patient is to have it off at once. How shall he tell her? "Well, you know, Mrs. Jones, this is not a great deal," and the poor woman's face lights up with a sigh of relief, which makes the doctor's task harder still, for how can he dash her hopes to the ground? "It certainly is a lump, and you would be better without it; but it is nothing of any very great consequence. Get Mr. So-and-So, the surgeon, to look at it; and if he thinks it had better come off, well, we'll soon manage that. An operation? Well, yes, if you like to call it so; but it's a very slight one. Nurse? Oh, well, yes, it would be, perhaps, as well to have one; but your daughter here will be able to do nearly all you'll want. You won't want much nursing. We'll soon have you about again." The time comes. A nurse has been engaged; the daughter, brave enough up to a certain point, dissolves into floods of tears at the critical time, and it becomes necessary to exclude her from the bedroom, lest her tearful, grief-stricken face should frighten her parent out of any little pluck she has. The nurse finds that she has all to do at the time of operation and afterwards. One finds the public generally full of sympathy for the patient and her relatives, especially if they "bear up wonderfully"; but it is not nearly so usual as it should be for them to consider the tired condition of a nurse after even a "comparatively small" operation. To such may be commended a perusal of the second paper in this little series of mine, which, although intended as instruction to nurses themselves, may serve to give outsiders some idea of the work required from a nurse only in preparation for a properly conducted operation in these aseptic days. I can assure them that the amount of work required from a nurse during the operation also is enough to tire most women. But many people expect them to be as spry as ever afterwards, as though they had been doing nothing. Many nurses can and do go on all through the day, but when it comes to expecting her to go on through the night also, even in the interests of the patient alone it is time to protest. But in the case supposed above what else is to be done? The daughter is useless; there is nobody else; the nurse pluckily volunteers. What wonder if exhausted nature is too much for her, and about two a.m. she drops off to sleep, whilst the patient soon after wakes and cries for a drink. Should the nurse's sleep be sound, and some relative of the family hear the patient's cry before her, the remarks of that relative the next day would induce any outsider to imagine that the nurse had been guilty of a deliberate attempt upon the patient's life. I remember one patient I had whose sister used to tell the nurse in an imperative tone, before going to her own bed, "Now, nurse, I am sleeping in the next room, and I am a very light sleeper. I shall know immediately if my sister has to call in the night, and I shall be in here directly." Now this was not merely offensive—some people cannot help being offensive—but totally unnecessary, since the nurse to whom it was addressed was a specially good one—and there were two (a night nurse and a day one), so she was not overworked. Such speeches are, I am glad to believe, rare; but they do occur sometimes, and they do not tend to increase the nurse's devotion to the particular patient in whose interests the speakers is presumably talking. Novel writers also are exceptionally fond of writing about nurses as though they possessed a supernatural fund of strength and wakefulness. Here is a quotation from the first I pick up: "Lady Emily, indeed, clung to her (the nurse) piteously when she reappeared at the bedside. She must stay, she told herself, at any rate until the worst was over. For three days and nights"—the favourite time—"Sister Christina never left her. She would snatch an hour's sleep now and then on a sofa in a corner. . . . Then, when there was a sudden and definite change for the better in the patient, it was the nurse who broke down." As the physician drives away from the house with the nurse, whom he is taking home to his own house to be nursed herself by his wife—physicians in novels have, apparently, nothing else to do with their houses than use them as hospitals for sick nurses, and their wives no other aim in life than to act as matron—he turns to the master of the house, and says, "You must thank this young lady, Mr. Laidlaw, for the prospect of many years of future happiness with your amiable and accomplished wife. In some cases we doctors can do little; the nurse does everything. Skill, sympathy, and untiring care—these, I may say, are eminently the qualities possessed by Sister Christina. If our dear Lady Emily is, happily, now alive, it is entirely due to her devotion."

Now, arrant rubbish as this is, it yet has some importance, because even novelists reflect, to some extent, the current views of life; and so many foolish women read this sort of stuff, and are bitterly disappointed when they find that the Sister Christinas of everyday life have more common sense than to play the sentimental idiot after this fashion. Consider the plain facts. Illness of whatever kind is a serious thing. It is understood by doctors and nurses, but it is not by the general public. The public therefore engage doctors and nurses to do for them the things necessary for their return to health. These things are very important, and their importance extends also to the way in which they are done. After an operation, for instance, there are many things to watch for, and to do, without hesitation and without flurry—to be done neatly, quickly, and smartly. They cannot be done safely and so as to serve the interests of the patient if they are taken in hand perfunctorily, stupidly, or by a person worn out by fatigue and want of sleep. The public wants, and ought to have, at such a time, persons near them who are able to give them the smartest service possible. What men or women are at their best for twenty-four hours running? In order to obtain such best service possible, and which here is the only service worth having at any price, you must arrange for recuperation, or your nurses will be only able to give you their second or third best, which, in a case like this, is often worth nothing at all. Of course, if all you want is a sloppy, sentimental idiot who can do nothing at a pinch but cry, or proffer a useless sympathy, it does not matter whether she is half-asleep or snoring; but that is not a nurse, whatever she may call herself. Unfortunately, many people still retain the old ideas that illness and its like is a judgment from Heaven, under which everybody is equally powerless, and that all that can be done is to sit down and wail together, waiting for some supernatural power to remove the trouble which it has sent. Naturally, to anyone who really believes this, the sympathising clasp of some other person's hand represents the extremest help and comfort possible on this earth; and the only thing that person need do is to keep just so much awake as to be able to return their pressure or to hand them a drink.

Do not let me be misunderstood. The ultimate result in any case is in higher hands than ours. But the more we learn the more we find that that higher Power has allowed us to see to some extent the laws which operate here. We realise that many of our physical troubles are due to ignorant transgression of those laws—that persistent transgression, although merely ignorant, leads direct to death—that in many cases accommodation to those laws, and work in accordance with them, leads back to life and health; that the more we learn of them the greater power we possess; that by accumulation of such knowledge many things are now possible which formerly were not. It is only necessary to mention typhoid fever and small-pox on the medical and aseptic surgery on the surgical side to prove this; and the progress shown in mass by these things is no less real in detail in any individual case; so that nowadays a real nurse is someone who can do more for her patient than merely sympathise with her, or "smooth her dying pillow"—she is often a very potent factor for good or evil. To obtain the best work from her, then, she must be wideawake, alert, ready, and this is simply not possible unless she has regular food, regular exercise, and regular sleep.

Now it is scarcely necessary to do more than to point this out; all sensible persons will see the reasonableness of it, and will recognise that it is in their own interest that such arrangements should be made. It is the most short-sighted policy to treat operations, however simple, as things to be hurried over, scrambled through anyhow, or made light of in order to deceive nervous people into a kind of fools' paradise. And it is the same with illness, though a surgeon like myself would prefer that that side of the question should be dealt with by a man who makes medical matters his special care.

It may be thought that I am speaking with brutal frankness. It may be said that such plain speaking is unnecessary, since no one does make light of such matters, but I am not without actual experience to justify what I have said.

It is very frequently necessary to speak very distinctly as to the absolute necessity of treating nurses, not as machines, nor yet as angels, but as skilful, kindly human beings, whose help is valuable, most valuable, because backed by knowledge, and of a kind absolutely undreamt-of by the Betsy Prigs and Sairy Gamps of our fathers, but who have all the failings of our common humanity, and who are quite as liable to be tired out as the lady who engages them, or of being muddled and rendered irritable for want of fresh air and exercise as the master of the house in which they happen to be.

### For Reading to the Sick.

### DIVINE SYMPATHY.

### Verses.

How was He,
The Blessed One, made perfect? Why, by grief—
The fellowship of voluntary grief—
He read the tear-stained book of poor men's souls,
As I must learn to read it. —*Kingsley.*

If one heart in perfect sympathy
Beats with another, answering love for love—
Weak mortals, all entranc'd, on earth would be,
Nor listen for those purer strains above. . . .
Thou know'st our bitterness! Our joys are Thine!
No stranger Thou to all our wanderings wild!
Nor could we bear to think how every line
Of us—Thy darkened likeness and defil'd—
Stands in full sunshine of Thy piercing eye,
But that Thou call'st us brethren! Sweet repose
Is in that word; the Lord Who dwells on high
Knows all, yet loves us better than He knows.
—*Keble.*

Awhile ago I passed
Where every step seemed thornier and harder than the last;
Where bitterest disappointment, and inly aching sorrow
Carved day by day a weary cross, renewed with every morrow—
The heaviest end of that strange cross I knew was laid on Thee;
So I could still press on, secure of Thy deep sympathy.
—*F. R. Havergal.*

O blessed Lord, the thought of Thee,
When clouds our fairer vision mar;
When we are not where we would be,
And dearest friends are set afar;
The thought that 'tis Thy ruling will,
The thought that Thou art with us still,
Nearer than ear or eye can know,
And with us still in life or death,
In blooming life or failing breath,
'Tis all of Heaven we need below.
—*Isaac Williams.*

O blessèd voice of Jesus; which comes to hearts opprest!
It tells of benediction, of pardon, grace, and peace,
Of joy that hath no ending, of love which cannot cease.
—*Dix.*

### Reading.

"For we have not an high Priest which cannot be touched with the feeling of our infirmities; but was in all points tempted like as we are, yet without sin."—*Heb.* iv. 15.

We never feel the comfort of sympathy more than in times of sickness or sorrow. It is pleasant to have those around us who take part in our joys; but it is more valuable to meet with a fellow-feeling in our troubles. Some sick people have to bear their sickness all alone; no one comes near them, except now and then! none but strangers. And yet even they have a Friend near—the Presence of God. I could not speak to Him so freely if He were not near. It is no presumption to say "the Father is with me." My heart tells me it is true. It is the witness of the Spirit. I do not deceive myself in this, for God promises His Presence, and that which I feel is but the fulfilment of His promises. "Draw nigh to God," St. James says, "and He will draw nigh to you." What is this but His Presence? And our Saviour Himself gave a yet more definite promise, "If a man love Me he will keep My words; and My Father will love him, and We will come unto him, and make our abode with him." That hope in Thee, which Thou hast given me, not all the world should tempt me to give up; and I do from my heart desire to do Thy will, and to keep Thy Word.—*Francis Bourdillon.*

### A Book and its Story.

### ETIDORHPA.*

SUCH is the title of the book which lies before us. We have given it in full because it tells so much about the most remarkable book we have seen for many a day. One might sum up its characteristics as combining the methods of Jules Verne with the mysticism of Swedenborg; it is all so impossible, from the point of view of experience, yet so detailed is the narrative, so elaborate the scientific explanations, that it is equally impossible when reading it to refuse credence to the tale. The hero, who tells his strange experiences to Llewellyn Drury, is known to the reader only as "I-am-the-Man-Who-did-it." What he did was against his own will. Convicted of betraying the secrets of a mysterious society to which he belonged, he was taken away from his home, changed in appearance and even in voice from a young man into an old one by the action of chemicals unknown to ordinary science, and sent on a mission to the interior of the earth. What he saw there forms the subject matter of the tale.

The Man—we must for convenience shorten his title thus—made his descent at the famous Mammoth Cave of Kentucky, and the blind fishes of that famous cavern seem to have given the idea for the description of his guide: "He was less than five feet in height. His arms and legs were bare, and his skin, the colour of light blue putty, glistened in the sunlight like the slimy hide of a water dog. He raised his head, and I shuddered in affright as I beheld that his face was not that of a human. His forehead extended in an unbroken plane from crown to cheek-bone, and the chubby tip of an abortive nose without nostrils formed a short projection near the centre of the level ridge which represented a countenance. There was no semblance of an eye, for there were no sockets. Yet his voice was singularly perfect. His face, if face it could be called, was wet, and water dripped from all parts of his repulsive person. Yet, repulsive as he looked, I shuddered more at the remembrance of the touch of that cold, clammy hand than at the sight of his figure, for a dead man could not have chilled me as he had done with his sappy skin, from which the moisture seemed to ooze as from the skin of a water lizard."

It says much for the power of the author that he represents the Man as becoming attached to this unattractive personage, and that we do not wonder at it. The general impression of the interior of the earth is that it is a central fire. The Man did not find it so. On the contrary it is a land of infinite variety; and it differs from the surface only in that gravitation and other natural laws which we assume to be universal are there non-existent. It is illuminated by the light which the earth absorbs from the sun, and which is diffused by passing through the crust of "space dust" with which we are familiar. A gentle heat is obtained from the same source. Air might be expected to be lacking, but we are assured that as much as is necessary filters through cracks in the earth crust. But, indeed, much is not needed, for with the suspension of all the ordinary laws of nature, the Man can live without the effort of breathing. His guide even reckons up with him the amount of life energy which is used up in the mere effort of supporting life. At first he is fed with slices of wonderful fungi, which possess the flavour of pineapple, strawberry, and all other delectable things; but in the end he is so etherealised that hunger, like all other mortal attributes, is lost. Each step he takes carries him yards on his way; he leaps over precipices and lands thousands of feet below as lightly as a feather. He sails with magic speed across a lake, whose rare but periodic overflow causes a volcanic eruption on the surface of the earth; and when Llewellyn Drury, listening to the strange story, expresses doubt of this or that miraculous experience, the Man is at the trouble of showing him by experiment on a small scale how all that he describes is possible on a larger one. To us these explanations are the most fascinating part of the book. We long to go to the laboratory of a physicist to test for ourselves the truth of the Man's statements. It is all convincing, as convincing as any of Robinson Crusoe's experiences, while at every turn we also, with Llewellyn Drury, refuse to believe it.

But this story of adventure is only the framework for a system of mystical morality which it is more difficult to summarise. Indeed, we do not feel able to attempt the task. The guide tells of environing spirits, of a deity whom our earth-knowledge cannot comprehend, of powers within our reach yet out of it because of our material conception on all questions. The author has an intense hatred of cruelty, which goes so far as to make him devote one whole chapter to the denunciation of the study of biology. In this chapter one part is, or is pretended to be, excised, and only by the context left do we gather that it is a ghastly tale of an anatomist who sacrificed a living child to his passionate desire for knowledge. In this side of the story, too, there is the same earnestness, the same power of carrying conviction to the reader which characterises the merely marvellous portion.

But what is the meaning of the title, "Etidorhpa"? Etidorhpa is the name of a beautiful maiden whom the Man meets while undergoing temptation in the "Drunkard's Den." She explains that she is the spirit of love (and the astute reader may note that the name is "Aphrodite," spelt backwards). Whether or not there is any mystic meaning in this reversal, it is certain that Etidorhpa shows the dual nature of love. She tempts the Man with the drunkard's cup, yet weeps as she sees him stretch forth his hand to take it; and it is the thought of her which enables him to withstand the temptation. The scene in which she first appears is grotesque; it belongs, indeed, to the realms of the Christmas pantomime. The drunkards are like the gnomes and other servants of the wicked giant. Their most obvious characteristic is that one feature has absorbed the energy of all. A great hand, with an enormous pointing forefinger, is attached to a tiny body, and so forth. After this comes the ballet—Mr. Lloyd must forgive us the phrase; it seems to us the only appropriate one—in which Etidorhpa appears. But with her appearance the dignity of the book, for a moment impaired, is at once recovered. "My name is Etidorhpa," she says. "In me you behold the spirit that elevates man, and subdues the most violent of passions. In history, so far back as to be known as legendary mythology, have I ruled and blessed the world. Unclasp my power over man and beast, and while heaven dissolves, the charms of Paradise will perish. I know no master. The universe bows to my authority. Stars and suns enamoured pulsate and throb in space and kiss each other in waves of light; atoms cold embrace and cling together; structures inanimate affiliate with and attract inanimate structures; bodies dead to other noble passions are not dead to love. . . . Take from the life of man the treasures I embody, and he will be homeless, childless, loveless. The thought of heaven will in such a case be as the dismal conception of a dreary platitude. A life in such a heaven, a heaven devoid of love (and this the Scriptures teach), is one of endless torment."

This is a unique and memorable book. The schoolboy may read it for its adventures, the scientist for its bewitching suggestions of unknown laws and powers, the theologian for the moral elevation of its thoughts, and each will return to it again with renewed interest.

* "Etidorhpa; or, The End of Earth. The Strange History of a Mysterious Being, and the Account of a Remarkable Journey. As communicated in manuscript to Llewellyn Drury, who promised to print the same, but finally evaded the responsibility, which was assumed by John Uri Lloyd." With many illustrations by J. Augustus Knapp. Eighth Edition. (Cincinnati: The Robert Clarke Company. 1897.)

### Everybody's Opinion.

[Correspondence on all subjects is invited, but we cannot in any way be responsible for the opinions expressed by our correspondents. No communication can be entertained if the name and address of the correspondent is not given, as a guarantee of good faith but not necessarily for publication, or unless one side of the paper only is written on.]

### THE TRIALS OF A DISTRICT NURSE.

DORA H. writes: Will any district nurse kindly let me know how long she remains with a midwifery case after it is all over and both mother and baby doing well, as doctor I work for thinks the nurse should stay ten or fourteen hours? How they think the other patients are going to do I cannot say. In my district they think the nurse should do day and night duty and never grow weary or be ill. All the doctors want you first, and think you should go first in their case. So I should like to know how other nurses manage?

### A CANCER CASE.

"H. M. S." writes: Seeing that "M" in so kindly sending information for the correspondent who has a cancer case mentions carbolised tow as being 1s. per lb., thinks it may be worth mentioning that Messrs. Maw, Son. and Thompson list it at 8d. per lb., also the tarred tow, the odour of which is more acceptable to some; and in the same list peat moss fibre is down at 1s. 8d. per lb. "H. M. S." has bought the carbolised tow repeatedly of local chemists for 8d. the lb., and the unmedicated for 6d., this latter being cheaper than the oakum spoken of by "M." as being 7d. from even a wholesale chemist. Doubtless, the tow could be had for less than 6d. wholesale. "H. M. S." has found it of great use in a very bad case of cancer.

### NURSING IN WORKHOUSE INFIRMARIES.

MARION writes: Doubtless those of your correspondents who were disposed to laugh at and distrust the efficiency of the querist in regard to bed sores regret their haste when they read of the difficulties which beset her. Pressed by duties and goaded by worries, she must fight against odds. Would it not be well if others holding similar positions would let your readers have their views on union infirmaries, their salaries and facilities. There is apparently a wide field for improvement in that branch of nursing. Trained nurses are now employed to do duties heretofore performed by the unskilled. Are they paid in proportion to their abilities? I fear not. I would like to see the subject freely discussed. Would you kindly afford space to my letter as I am much interested in this matter. Let "M. B." hope for better things and still keep "soft-hearted," knowing that what she does for the least of God's creatures is done for Him.

### SISTER MONICA.

"JUSTICE" writes: As no one has made any comment on the article in your paper headed "Sister Monica," I would like to correct the statement. Warwick is nursed by two Church of England Queen's Jubilee Nurses, who do all the general nursing. Sister Monica is the midwife employed by the ladies of a subscription association called the "Ladies' Charity." It is for poor women, and one of the stipulations is that the infants born are taken to be baptised as soon as possible after birth, and they do not get the full benefit of the charity until the children have been received into the Church, and the mother has also to be "Churched" herself. Perhaps, therefore, the objection of the ladies is not altogether unreasonable, and certainly Sister Monica is not the head of the Warwick Nursing Association, for it is quite and entirely a distinct thing.

*** "Justice" evidently did not see that we corrected the statement as to Sister Monica being head of the Warwick Nursing Association in our next number. We are glad to receive the information as to the rules of the lying-in charity. —ED. *T.H.*

### THE TRIALS OF A MONTHLY NURSE.

"A PERPLEXED MONTHLY NURSE AND L.O.S." writes: Will you kindly tell me the usual custom in monthly nursing as regards "off-duty" time? I have been told that it is not customary to go out for the first ten days, or even three weeks, which would seem to be an almost impossible arrangement for a busy nurse often going straight from one case to another, as her health and, indirectly, her patient, would suffer. Half an hour even a day in the immediate vicinity would be acceptable. I should be glad also to know what is the shortest "general" or surgical training for which a certificate is given to a paying probationer and the name of the hospital giving it; and, if I have not already trespassed too far, I would like to ask whether the L.O.S. is a certificate or a diploma, and if the nurse gaining it is recognised as a midwife, having as much right to practise as such as she who holds a midwifery and monthly nursing certificate from one of our large lying-in hospitals; or does any trained nurse undertake midwifery at her own risk, as it were? I have recently seen in the "Mirror" the L.O.S. alluded to as a diploma, but a doctor has told me—whether from medical prejudice or not I do not know—that it was not a diploma, and that I had better put nothing about it on my cards, as it might influence medical men against me. On the other hand, a very clever doctor in the same place told me that it was well thought of, and showed the possessor must know her duty.

[A reasonable amount of daily exercise is necessary in all branches of nursing. Unless complications arise which forbid the patient being left for an hour, a nurse should arrange to spend that time in outdoor exercise. She should consult her patient's convenience in fixing the time. The London Obstetrical Society gives a certificate of fitness for the work of a midwife, which certificate is the highest a nurse can hold, and makes her a midwife. The attitude of different members of the medical profession towards the holders of it varies considerably. A midwife who confines herself to her own work, and sends for the doctor when necessary, is soon appreciated by those who understand how great the value of skilled help is and the danger of ignorance in this branch of the work.—ED. *T.H.*]

### A DISTRICT NURSE'S PAY.

"ONE OF THE SECOND THOUSAND MEMBERS OF THE PENSION FUND" writes: I think F. Taylor has mistaken her vocation in choosing nursing as her profession. In what work is there greater need for a cheerful disposition than among the sick? I speak of cheerfulness as distinct from levity of manner. Having been engaged in the work since 1875, and had a great many hard, trying cases, I know well the feeling of weariness that oppresses us, and that sometimes causes us to feel our work a burden. But, at such times we can appreciate the blessing of having health and strength to attend others (many of whom suffer long periods of pain and weakness, with no prospect of restoration to perfect health), instead of ourselves being laid aside in their helpless condition. I do not agree with F. Taylor that it is a woman's first duty to try and get married; but it is the duty of every individual to discharge his or her duties in their state of life to the best of his or her ability and for the good of others. I think we have a great many privileges which our married sisters do not possess; we escape many cares and anxieties and troubles which fall to their lot. We each of us have some work allotted to us, and, as the poet says:—

"She who her destiny strives to fulfil  
Will leave her affairs to His Sovereign will  
Who guides each event in her life;  
And then a rich blessing she surely shall prove  
To all who shall bask 'neath the rays of her love,  
As maiden, as mother, or wife."

I should advise F. Taylor to acquaint herself with the working of the Pension Fund—by reading "Ministering Women" or studying the prospectus of the Fund. We owe a debt of gratitude to our esteemed founder and to those who have so generously helped him in establishing and bringing it to its present position of success. That they may long be spared to rejoice in the result of their labours is the desire of "One of the Second Thousand Members."

### The Victoria Commemoration Club,

29, SOUTHAMPTON STREET, STRAND.

### COOKERY FOR NURSES.

MISS HELEN THOMSON announces that, at the request of many nurses and members of the club, she has arranged to give private practical cookery lessons in the club kitchen. These lessons will be given at any time to suit the individual nurse, and she may also choose the dishes she wishes to learn. The charge for each lesson is 4s. The materials used will be provided by the club, and the dishes when made to be the property of the club, and sold in the dining-room after each lesson

### Notes and Queries.

*Comparisons.*

(145) I am desirous of becoming a probationer in some good training school, either in London or Dublin, and shall be extremely obliged for information as to the value a certificate (three years) obtained in Dublin at the Adelaide, Meath, or City of Dublin Hospitals, as compared with a certificate obtained in one of the smaller London hospitals, for example, King's College, St. George's, or Charing Cross?—*A Candidate.*

At the London hospitals you mention a three years' course of instruction in the wards is necessary, at those in Dublin one year's training in the wards is considered enough, the training during the subsequent voluntary years being given in private work. The value of a certificate is to be measured by the sort of appointments for which it qualifies, and the action of the Local Government Board in demanding a three years' certificate for all its higher appointments gives the official stamp to the system of training for three years. Therefore there can be no question as to which is the most valuable. "A Candidate" would be able to form her own opinion if she consulted Honnor Morten's "How to Become a Nurse," price 2s. 6d., of the Scientific Press, 28 & 29, Southampton Street, Strand.

*Agreement.*

(146) I have signed an agreement to stay with a Nursing Association for three years. Since I have been here the secretary has heard a "rumour" that I intend leaving before that time, and she has stopped paying my salary until she has £6 10s. in hand. Is it legal for her to do so? I have no intention of leaving, as, if I did so, I should forfeit my certificate, and I have only myself to depend on. I have now been here twelve months.—*E. S.*

An agreement is equally binding on both parties signing it, therefore the association is legally bound to pay Nurse S. her salary on the dates fixed by it.

*Superfluous Hair.*

(147) Could you or some of your correspondents kindly tell me a remedy for destroying superfluous hairs? It is a most delicate matter, I know, but a sister of mine is very disfigured with them on her face. She has spent a great deal in trying different remedies; they only seem just to burn them away, not to touch the roots at all. She is very troubled about them, as children have been heard to remark about them. I know several ladies who suffer the same, so perhaps by sending a remedy through your paper you would help several.—*Nurse Gertrude.*

Nothing permanently removes superfluous hair but destroying each root separately by electrolysis. It is a troublesome and long operation, but entirely successful. Medical men who give special attention either to skin diseases or to electrical treatment would be able to give relief.

*Body for Sale.*

(148) Would you be so kind as to tell me the best way to dispose of my body for dissecting purposes, when dead, to a hospital, and about the price they give for one? as I am hard up for money, and if I can obtain a reasonable price for my dead body I shall take it.

As the law stands at present we doubt whether a still living body is a very valuable asset. There are so many circumstances in which the coroner might intervene that the security offered is not of a very high character. Still, if our correspondent is suffering from some mortal disease which is likely soon to terminate in death, it is just possible that by applying to the dean of some medical school some arrangement might be made, but the price offered would be a very small one, because practically there is no security, and nothing to bind the relatives.

*Death Certificate.*

(149) Will you kindly tell me if certificated midwives can give certificate for burial of dead-born infants?—*Nurse P.*

A certificated midwife cannot give a certificate for the burial of a still-born child. She may in such cases have to sign a declaration (if she were present at the birth), stating that no registered medical practitioner was present at the birth, or that his certificate cannot be obtained, and that the child was born dead.

*Books for Mothers.*

(150) I should be so glad if you would tell me as soon as possible the name of a good, simple, inexpensive book for mothers concerning the care of their children; (2) also the name of a book for young girls or young women on the care of their own health.—*"Tryon."*

"The Mother's Help, a Guide to the Domestic Management of Her Children," by P. Murray Braidwood, M.D., price 2s., from the "Scientific Press," 28 & 29, Southampton Street, Strand, London, W.C.

*Homes for Incurable Consumptive Patients.*

(151) Would you kindly tell me where there are homes for phthisical patients, and how to set about getting a girl into one of them? She is absolutely homeless and friendless, and funds will not admit of much payment with her.—*Nurse H.*

There are a few. Home for Consumptive Females, 57 and 58, Gloucester Place, Portman Square, W. (Secretary, Walter Davies, 6, Waterloo Place, S.W.), entrance fee £1 1s., terms 7s. a week; St. Catherine's Home, Grove Road, Ventnor, terms by selection, and payment of 10s. 6d. a week. See also "Burdett's Hospitals and Charities," List of Institutions for Chronic and Incurable Patients, pp. 906-912.

*Training an Obstacle.*

(152) In December, 1895, having a wish to become a nurse, I joined a Cottage Nursing Association. My time will be up in June of this year. I am now desirous to become a probationer in a London hospital. Having seen the matrons of most of them I find that my previous training is objected to. Can you tell me what I had better do. I hold the L.O.S. certificate?—*M. R.*

We should advise "M. R." to obtain a copy of Honnor Morten's "How to Become a Nurse," price 2s. 6d., the Scientific Press, 28 & 29, Southampton Street, Strand, London, W.C., and write to the matrons of the hospitals she has not seen. If she fails, then she might try to obtain admission into a provincial hospital, or one of the larger Poor Law infirmaries, but if she is suitable in other respects we feel sure that she will find some who will give her the desired opportunity.

*Ergot.*

(153) Having attended two courses of lectures on midwifery at St. Mary's Hospital, Manchester, and received a diploma for the same signed by Drs. Lloyd Roberts, Walters, and Donald, with permission to give ergot in 30 drops to the dose if required after delivery, one doctor here disputes my right to use it. Please state if I should discontinue the use of it.—*One in Suspense.*

"One in Suspense" has exactly the same right to give ergot as any man or woman in the street has—neither more nor less. The certificate or "diploma" given by the authorities of St. Mary's Hospital gives no rights of any sort. As the law stands at present, if a midwife is assisting a qualified practitioner she must do as she is told. The practitioner, however, will be liable to be struck off the register for employing an unqualified assistant, except under direct personal supervision. If, on the other hand, she is acting on her own behalf she may do exactly as she likes; for aught we know she may do symphysiotomy or cæsarian section; so long as the patient gives consent there will be no penalty until something goes wrong, and then she will have to take her chance before the coroner. In regard to ergot, the midwife might, perhaps, come under the Apothecaries' Act if she gave it to save the patient's life, because then we might presume that the patient was ill, and it might be held that in giving the ergot the midwife was supplying medicine for the treatment of disease, and thus was acting as an apothecary. But if she gave it as a mere matter of routine at the end of labour she would probably be free from the operation of the Act. All this is very absurd, but then the existing state of the law about the practice of midwifery is entirely absurd. We should advise "One in Suspense," if acting under a doctor, to do as he tells her, and if acting by herself to send for a doctor if ergot seems required.

*Mental Disease.*

(154) I am anxious to buy some works which would be useful to me in the study of mental disease, being much interested in the subject. Could you inform me in "Notes and Queries" column of your paper whether any of the following would be likely to be useful: (1) Dr. Clouston's "Neurosis of Development," (2) Dr. Ireland's "Blot upon the Brain," or (3) Dr. Strahan's "Marriage and Disease"?—*Emilie.*

These books are perfectly appropriate for those who wish to study the questions discussed in them, but they are not books on the general question of mental diseases. One of the best general descriptions of insanity, in a brief form, is that contained in Bristowe's "Medicine." The articles on insanity in Quain's "Dictionary of Medicine" are also good, and we fancy this work can be seen at free libraries. A very good "Manual of Mental Diseases" is that by Campbell Clark, which was reviewed some time ago in THE HOSPITAL.

*Certificates for Nursing the Insane.*

(155) Will the Editor kindly give names of lunatic asylums where they give certificates for proficiency in nursing the insane?—*A. Kibblewhite.*

No asylums give certificates for proficiency in nursing the insane, but many asylums encourage attendants to take the certificate of the Medico-Psychological Association (for particulars of which write to the Secretary, 11, Chandos Street, W.). The Northampton County Asylum at Berrywood, near Northampton, is a noted one, but as the list of asylums in England and Wales occupies from page 592 to page 600 in "Burdett's Hospitals and Charities," it is impossible to give it here.

*Linen.*

(156) Would you kindly, through the medium of your columns, (1) tell me if household linen should be marked on the right hand side? (2) What books would you suggest as most useful from which to give courses of lectures to nurses? and (3) Where Miss Isabel Hampton's book is procurable; and would I be permitted to look over the Victoria Commemoration Club Rooms before becoming a member, as a constant subscriber to THE HOSPITAL?

1. Linen is marked in the top left hand corner. 2. "The Matron's Course," by Miss S. E. Orme, price 1s., from the Scientific Press, 28 and 29, Southampton Street, Strand, London, W.C., might suit you. 3. The same publishers would also get you Isabel Hampton's book for 7s. 6d. The secretary of the Victoria Commemoration Club is always glad to show bona fide visitors over it, but the readers of THE HOSPITAL have no special privileges.

*Training in Children's Ward.*

(157) My year's training will be finished in August, and I am very anxious to try and get a year's training in children's nursing only, but I am afraid I am too old to enter a children's hospital. Could you kindly help me through your paper, the "Nursing Mirror." My age is 32. Would you be so kind as to let me know what you think about advertising in your paper, the "Nursing Mirror." Could I do so without putting in my address?—*Nurse Gillies.*

Your best plan is to watch the advertisements in the "Mirror." There will probably be several suitable for you before August. If you do not find exactly what you want it will certainly be advisable to advertise yourself, in which case you should write to the Manager.

*Asylum Matronship.*

(158) I should be greatly obliged if you could inform me if a three years' hospital certificate is necessary to obtain an asylum matronship. Could such a post (in a small private asylum) be got with only the certificate of the Medico-Psychological Association and eighteen months' hospital training, but no certificate?—*Louie.*

Probably your training is enough for the post you seek. Do you know the "Asylum News," published at the County Asylum, Lancaster? This publication would probably be useful to you in the matter.

*Salaries of Storekeepers.*

(159) Would you kindly tell me what are the salaries of storekeepers in public institutions?—*Dorothy.*

They vary with the institution, as do the salaries of all posts.

*Children's Hospitals.*

In reply to query 123 in the "Nursing Mirror" of January 15th, Miss L. B. W. would like to state that the Alexandra Hospital for Children, Queen Square, Bloomsbury, is most in want of funds, as, being a special hospital, it is often overlooked.

## Figures and Tables

**Figure f1:**